# Knowledge hiding in teachers of moral education degree programs in Pakistan: The role of servant leadership, psychological ownership, and perceived coworker support

**DOI:** 10.3389/fpsyg.2022.860405

**Published:** 2022-09-09

**Authors:** Saima Anwaar, Liu Jingwei

**Affiliations:** ^1^Marxism College of Northeast Forestry University, Harbin, China; ^2^Heilongjiang Provincial Think Tank for Ecological Civilization Construction and Green Development, Harbin, China; ^3^Marxism College of Shanghai Lixin University of Accounting and Finance, Shanghai, China

**Keywords:** servant leadership, psychological ownership, perceived coworker support, Knowledge hiding, moral education

## Abstract

The purpose of this research is to examine the influence of servant leadership on teachers of moral education degree programs in Pakistan. By utilizing social learning, we propose that servant leadership and perceived coworker support can reduce the knowledge hiding by enhancing the sense of organization-based psychological ownership. The findings of time-lagged and multi-source data indicate that servant leadership has a negative relationship with knowledge hiding. Our results also indicate that psychological ownership mediates the effects of servant leadership on knowledge hiding. Moreover, a higher level of perceived coworker support enhances the sense of psychological ownership which helps to reduce knowledge hiding. This research extends strong support for the proposition that servant leaders who adopt an employee-centered management approach, stressing personal integrity and care for employees significantly affect employee attitudes and behaviors. Moreover, this study suggests that managers should demonstrate care toward their subordinates which helps them to reduce negative behaviors (e.g., knowledge hiding).

## Introduction

Knowledge management as a competitive advantage has recently gained the attention of researchers in academic studies (Adhikari, 2010; Latilla et al., 2018). Organizations are now well aware of the importance of knowledge in organizational success and they are adopting strategies that include leaders behaving as leader-cum-colleague (Amin et al., 2019), task-oriented activities (Abbas et al., 2020), creativity facilitating work environment, openness, safety, trust, and autonomy (Moll and Kretzschmar, 2017) to enhance knowledge sharing among employees. They are creating ways for organizing knowledge effectively so that a sustainable competitive advantage can be acquired (Hila and Shobaki, 2017; Jit et al., 2017; Amin et al., 2019; Abbas et al., 2020; Aboramadan et al., 2021). Given the above, institutions are trying their best to enhance the knowledge exchange behavior among employees by minimizing knowledge hiding intentions of the employees because they understand that knowledge sharing (Srivastava et al., 2006; Ansari and Malik, 2017; Amin et al., 2019; Gagné et al., 2019) is a key component in knowledge management system (Abdillah et al., 2020). Knowledge hiding attitudes of employees (Connelly et al., 2012; Bogilović et al., 2017; Ghani et al., 2020; Zutshi et al., 2021) can sabotage all the efforts of an organization toward knowledge management. This is a serious concern in recent managerial practices that despite investing a huge amount of resources to discourage knowledge-hiding behaviors, employees are still unwilling to share knowledge. Harmful consequences of knowledge hiding have been studied extensively (Bogilović et al., 2017; Butt and Ahmad, 2019; Arain et al., 2020; Butt, 2021). When institutions pursue knowledge-sharing practices, they discourage knowledge-hiding behaviors among employees. One of the major challenges in knowledge management is to design strategies for enhancing knowledge-sharing attitudes in employees by minimizing knowledge-hiding tendencies (Demirkasimoglu, 2015; Boz Semerci, 2019; Zutshi et al., 2021).

Knowledge hiding is “an intentional attempt by an individual to withhold or conceal knowledge that has been requested by another person” (Connelly et al., 2012). A survey carried out in the US reported that 76% of participants admitted that they had hidden knowledge at least once in some form or other (Connelly et al., 2012). According to Babcock (2004), knowledge-hiding incurs losses to the tune of US$ 31.5 billion in Fortune 500 companies (Wang and Noe, 2010). The negative outcomes as a consequence of the counterproductive behavior cost US$ 1 trillion; comparatively, it is US$ 120 billion for theft, US$ 4.2 billion as a result of workplace violence, and more than US$ 900 billion in income loss due to fraudulent activities (Banks et al., 2014). Knowledge hiding not only motivates employees toward counterproductive work behavior (CWB) but also prevents them from being creative. Despite efforts to enhance knowledge sharing in organizations, success has been elusive. It is becoming clear that in many instances employees are unwilling to share their knowledge even when organizational practices are designed for this. Knowledge hiding (KH) has also been reported as a serious concern in higher education institutes (Demirkasimoglu, 2015; Ghani et al., 2020; Zutshi et al., 2021) where knowledge sharing should be the core value of academia. Previous literature on knowledge hiding explored the several contextual factors that can prevent or encourage employee's engagement in KH behaviors (Bogilović et al., 2017; Khalid et al., 2018; Pradhan et al., 2019; Zhao et al., 2019; Guo et al., 2020; Koay and Lim, 2021; Syed et al., 2021).

Given the outlined KH phenomenon, we argue that leadership can play a vital role in developing the right attitudes among employees to hide knowledge less. Prior studies have pointed to abusive supervision as an antecedent to knowledge hiding (Khalid et al., 2018; Pradhan et al., 2019), but certain leadership styles that can prevent hiding knowledge are relatively less explored. Similarly, several studies have explored the psychological mechanisms to explain the relationship between organizational factors and knowledge hiding behaviors of employees. In the same vein, Jiang et al. (2019) explored the mediating role of psychological safety in knowledge hiding as a barrier to thriving, and Holten et al. (2016) reported the mediating effects of trust and justice on knowledge hiding. In the work of Riaz et al. (2019) the mediating role of job stress and occurrences of knowledge hiding and workplace ostracism was highlighted. Despite these several studies on different mediating factors in knowledge hiding, its prevalence in the context of higher education institutions in Pakistan still needs to be explored.

Drawing on the social learning theory, this research bridges the gap in the literature by exploring the mediating role of organization-based psychological ownership (PO) in servant leadership (SL) and knowledge hiding (KH) relationships. We argue that SL is positively linked with PO which further leads to the negative association with KH. Following the social learning theory, we point out that individuals learn from the behaviors of others and respond accordingly in a specific environment as they perceive others' behavior. We then connect this notion with our premise that ‘when leaders serve their followers in a very polite way and work with them as leader-cum-colleague, then they stimulate the prosocial behavior of their subordinates and help them out in enhancing their sense of psychological ownership. We propose that psychological ownership can play a role as a mediator between SL and KH.

Consistent with the outlined arguments and considering PO as an intervening variable between SL and KH, it is also essential for a thorough investigation of the boundary condition (moderator) in the proposed relation. Because it would be interesting to consider the boundary condition (circumstances) in which the relation of outlined variables may be affected in the presence of a moderator. Previous studies also focused (Malik et al., 2019; Syed et al., 2021) on different moderators on organizational factors and KH relationships. One recent study explored the moderating effect of individualistic and collectivistic values in perceived task conflict and KH behaviors (Boz Semerci, 2019). In this study, we propose that SL coworker support can interactively enhance the sense of psychological ownership which in turn can help organizations to reduce employees' KH behaviors. This study contributes to the current understanding of SL and negative employee behaviors and suggests ideas for managers and organizations to address this problem (e.g., KH). Based on social learning theory, we used organization-based psychological ownership (PO) as a mediator between SL and employees' KH. Our approach to examining the moderating role of coworker support as a boundary condition to the outlined relationship will also be a considerable addition to the literature related to educational institutions in Pakistan.

## Literature review and hypotheses development

### Servant leadership

In the current era, successful leaders are those that serve as role models for their followers, taking care of their subordinates, serving them with politeness, and creating a friendly environment. By coining the term “servant leadership,” Greenleaf made a purposeful attempt to change the direction of the organizational pyramid in the leader-follower relationship. He described it as “the servant leader is servant first. It begins with a natural feeling that one wants to serve, to serve first”. Despite several positive and negative reactions, servant leadership is a widely accepted approach in current management practices (Kumar, 2018). It guarantees a bright future in modern organizational practices being useful for the success of individual leaders as well as very purposeful for the organizations (Kumar, 2018). Servant leadership is becoming very useful in knowledge management (Andreeva and Kianto, 2012) where knowledge sharing plays an important role in managing the employees' skills and competencies (Cabrera and Cabrera, 2005; Bordia et al., 2006; Wang and Noe, 2010; Sharifkhani et al., 2016; Ansari and Malik, 2017; Gagné et al., 2019).

In addition, there are several specific instances where SL has emerged as an important means for institutions and organizations to counter knowledge hiding attempts of the employees. Several previous studies (Serenko and Bontis, 2016; Bogilović et al., 2017; Connelly et al., 2019; Zhao et al., 2019) have identified three major factors (organizational relations, individual traits, and knowledge contents) that influence the knowledge hiding behavior of employees. With regard to organizational relations, we considered SL as an influencing variable (predictor) for overcoming the negative outcomes of KH; previous research (van Dierendonck et al., 2014; Hila and Shobaki, 2017; Jit et al., 2017; Arain et al., 2020; Aboramadan et al., 2021) also endorse this. In this study, we propose that SL can be very useful in higher educational institutions to decrease employee KH behaviors. Drawing on the social learning theory (SLT) (Nabavi, 2012), we argue that when followers succeed in finding a leader who deals with great care and respect, then they try their best to respond in the same way. Because SLT suggests that behaviors of an individual are developed by observing the behaviors and actions of others. Therefore, when followers experience positive behavior from their leader then they respond in the same way and emulate the same level of care, love, and emotions. Studies have reported the positive outcomes of servant leadership. We argue that when servant leaders prioritize their subordinates and share their experience and knowledge with them, the subordinates also reciprocate in the same manner and avoid hiding knowledge. It implies that servant leadership can be an effective driver to minimize knowledge hiding in employees of higher education institutions given that KH can severely affect the overall organizational performance including the well-being of the individuals.

### Servant leadership and knowledge hiding

To the best of our knowledge, studies on servant leadership and knowledge hiding are limited, though KH has been studied along with other leadership styles (Abdillah et al., 2020; Guo et al., 2020; Koay and Lim, 2021; Syed et al., 2021). Yukl (2013) elaborated leadership as “the process by which a person exerts influence over others and inspires, motivates and directs their activities to help in achieving the organizational goals”. The study also indicated that leaders could influence their followers toward increasing their performance for the overall success of the organization. Similarly, the servant leadership style is very successful in earning followers' trust (Lee et al., 2018), since SLs also focus their efforts on ensuring the well-being of their followers (Connelly et al., 2019). They encourage, appreciate, support, praise, coach, and mentor their followers to enhance their performance. These kinds of leaders continuously gauge the performance of their followers so that it can be aligned with the organizational goals. In addition, servant leaders prioritize the needs of their followers and consider them while making decisions. This enhances employees' knowledge-sharing behavior and weans them away from potential knowledge-hiding attitudes.

Knowledge hiding discourages honesty, prioritizing and caring for others, ethical behaviors, and participation in the collaborative activities of the organization (Connelly and Zweig, 2015; Škerlavaj et al., 2018; Peng et al., 2020). It has a negative effect on individuals and organizations. Knowledge hiding is an intentional attempt by an individual to conceal or withhold knowledge that has been requested by another person (Connelly et al., 2012). It not only encourages counterproductive work behavior (CWB) but also prevents them from being creative. It may also negatively impact the creativity of the offender (Cerne et al., 2017). Knowledge hiding is not only harmful from an organizational perspective, but it also affects team performances along with individual performances (Arshad and Ismail, 2018).

Based on social learning theory, it can be proposed that servant leaders could be an efficient means of minimizing knowledge-hiding intentions among followers. Thus, we can argue that when servant leaders are polite, friendly, and calm, then followers learn from their behavior and respond in the same way. Likewise, when servant leaders treat their followers as colleagues and understand their needs, then the chances of negative attitudes (e.g., knowledge hiding) from the follower would be less. Thus, based on the above discussion we hypothesized:-

*Hypothesis 1: Servant leadership is negatively related to knowledge hiding*.

### Servant leadership, psychological ownership, and knowledge hiding

Servant leaders can build a very healthy, cooperative, and sound organizational culture (Sarkus, 1996). They earn employees' support on account of their reliability and commitment (Green et al., 2015). We argue that SLs can develop a striving-oriented environment by creating a sense of personal integrity and care for the followers, which in turn enhances their positive attitudes. In their study, Liden et al. (2008) reported the consistent efforts of servant leaders to empower, display accountability, create trust and establish ethical behavior while serving their followers. Consequently, such efforts influence employees and create a sense of ownership for their organizations. Organization-based PO means employees consider the organization as their own/personal organization and work for the organization in the same way. We argue that when leaders interact as sincere colleagues instead of reinforcing hierarchy and authority, then they can associate with their subordinates better by minimizing the power distance (Lin et al., 2018). Consequently, when employees have a sense of psychological ownership they display citizenship behavior toward the organization (O'driscoll et al., 2006). Similarly, leaders with a servant leadership style (Aboramadan et al., 2021) focus on the needs of their subordinates to stimulate positive employee behaviors. When employees receive support from their supervisors, it enhances their sense of organization-based psychological ownership. In addition, it is natural for people to be inspired by their role models and emulate their behavior. It is, therefore, asserted that when employees perceive that their leader is serving their needs their sense of psychological ownership will be improved.

Again, based on SLT, we postulate that the service attitude of servant leaders can motivate employees to be committed to the well-being of the organization by increasing their sense of organization-based PO. Because PO is an employee's positive attitude that is developed in the employees first by using best organizational practices, this PO can later play a vital in enhancing positive organizational outcomes (such as knowledge sharing) (Hameed et al., 2019). We argue that leaders as an actor can influence the employee's sense of organization-based PO. In this regard, SL is the best leadership style that can create a sense of PO among the employees. Although PO is a construct that has been widely studied in organizational contexts (Van Dyne and Pierce, 2004; Mayhew et al., 2007; Md-Sidin et al., 2009; Avey et al., 2012; Asatryan et al., 2013; Park et al., 2015; Dawkins et al., 2017; Kim and Beehr, 2017; Khatri and Dutta, 2018; Ali and Sagsan, 2021; Dahleez et al., 2021; Degbey et al., 2021), its implications and usefulness in the context of educational institutions and teachers have not been explored yet. In addition, its mediating role in servant leadership and knowledge hiding with special reference to teachers from higher educational institutions in Pakistan remains to be studied. Accordingly, we propose that when a servant leader is caring, loving, and friendly and prioritizes followers' needs, takes them into cognizance while making decisions, and addresses their problems and issues, then organization-based psychological ownership is developed among employees which can be used later for the betterment of the organization. When leaders respect their followers, then the followers reciprocate likewise as suggested by social learning theory. A feeling of ownership is developed in the employees because of servant leadership which is later used for prioritizing the organization's goals over personal goals. In line with this argument, we hypothesized: -

*Hypothesis 2: Servant leadership is positively related to psychological ownership*.

In the literature on servant leadership, several studies examine the relationship between servant leadership and employee behaviors and attitudes. Some studies have explored the antecedents of servant leadership such as conscientiousness (Krekeler, 2010; Hunter et al., 2013), agreeableness (Krekeler, 2010), and extraversion (Hunter et al., 2013), while others (Yildiz and Yildiz, 2015; Hila and Shobaki, 2017; Amin et al., 2019; Aboramadan et al., 2021) have investigated the consequences and outcomes of SL i.e., organizational citizenship behaviors, lead-member exchange, quality of service, employees satisfaction, job engagement, organizational commitment, organizational effectiveness, and organizational identification. In addition, some studies (Hunter et al., 2013) also explored the relationship between servant leadership and several negative variables i.e., disengagement and turnover intentions.

Using social learning theory (Bandura, 1985; Nabavi, 2012) as a theoretical construct to understand the functioning of servant leadership, we aim to explain the relation between SL, PO, and KH. Social learning theory (Bandura, 1985) provides an understanding of individual characteristics and behaviors that can create and influence the perceptions of the followers in a leader-follower relation. According to the theory, individuals learn from the behavior and attitudes of their role models and act accordingly as per the perceived relationship. Therefore, we argued that PO could be the possible antecedent of SL that consequently mitigates the effect of knowledge hiding by mediating the relation between SL and KH. Consistent with these arguments, leaning on the social learning theory and based on empirical studies discussed above, we hypothesized: -

*Hypothesis 3: Psychological ownership is negatively related to knowledge hiding*.

Several studies have explored the psychological constructs that explain the decrease in knowledge hiding behaviors in employees. For instance, Jiang et al. (2019) explored the mediating role of psychological safety in knowledge hiding as a mechanism to thrive. Holten et al. (2016) reported the mediating role of trust and justice in KH using a research model. Riaz et al. (2019) found the mediating role of job tension and sustainability in knowledge hiding and workplace ostracism. Reviewing other literature, we further proposed that PO can mediate the relationship between servant leadership and knowledge hiding. But more specifically, when servant leadership is high then it increases the feelings of psychological ownership in employees and this influences positive attitude in employees that further mitigates knowledge-hiding behavior of employees. Therefore, we hypothesized:-

*Hypothesis 4: Psychological ownership mediates the effect of servant leadership on knowledge hiding*.

### Moderating role of coworker support

Emotional support is a person's belief in their value, esteem, and care before someone else's (Amarneh et al., 2010). Social support may increase the well-being of an individual by influencing the relationship between the two constructs (Hamaideh et al., 2008). Studies revealed that social support can increase an individual's health and well-being (Hamaideh et al., 2008). Support from a loving person can minimize the level of stress and motivate an employee toward accomplishing any desired task (Hobfoll and Vaux, 1993). Previous studies have (Connelly et al., 2019; Anand et al., 2020) investigated (Malik et al., 2019; Syed et al., 2021) different moderators in leadership and KH relations. Boz Semerci (2019) explored the moderating effect of individualistic and collectivistic values in perceived task conflicts and KH behaviors while (Ghani et al., 2020) highlighted the moderating role of professional commitment in perceived interactional justice and playing dumb (type of KH) behavior.

**Figure 1:**
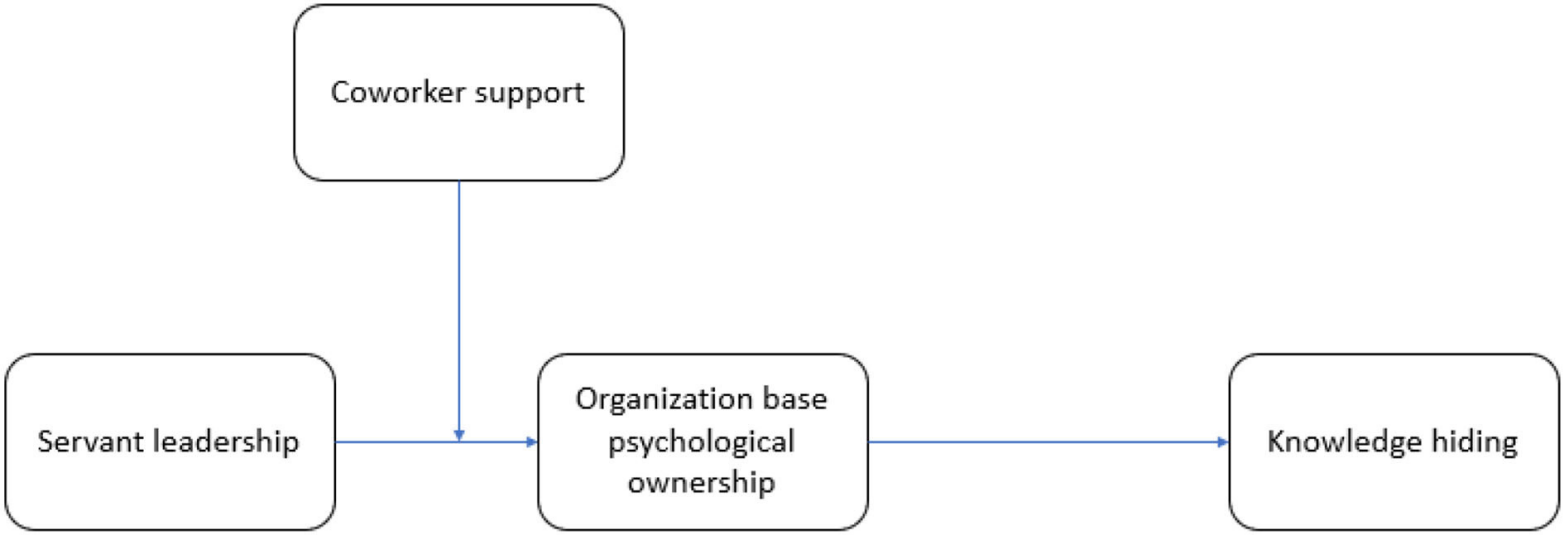
Research framework.

Pursuing social learning theory (Bandura, 1985), we considered coworker support as a boundary condition and propose that coworker support can moderate the relationship between SL and PO positively. Various studies have investigated the positive and negative effects of social relations in organizations (Hamaideh et al., 2008; Amarneh et al., 2010; Onderwater, 2017; Yoo et al., 2018). We added that the relationship between SL and followers' PO can be influenced by the moderating effect of coworker support and when coworker support is high/low then the relationship between SL and PO will be stronger/weaker. In organizational contexts, coworkers influence the relationship between the supervisor and subordinate or leader and follower. In the current scenario, we investigated the moderating role of coworker support in servant leadership and psychological ownership. We suggest that when individuals experience social support from their coworkers, their PO level increases. In particular, we hypothesize that coworker support may have a moderating effect on the positive relationship between servant leadership and organization-based psychological ownership of employees. The relationship between SL and PO will become strong in the presence of coworker support, and will be weakened in the absence of the same (see Figure 1): -

Hypothesis 5: *Coworker support moderates the relationship between servant leadership and psychological ownership, such that the relationship becomes stronger when coworker support is high*.

## Methods and procedures

After determining the suitable sample size and choosing appropriate measures, we proceeded toward quantitative data collection using the survey method. For data collection, we used the survey method as it constitutes a quantitative approach that allows researchers to witness the behavior (Rossi et al., 2013) and examine the association between variables. Using convenient sampling, we collected three rounds of data from teachers of moral education degree programs in higher educational institutions in Pakistan. The authors contacted sources in their targeted higher education institutions and requested permission to obtain data. We fixed an appointment with each source and request their help to ensure the availability of the colleagues. Using this procedure, we administered paper-pencil surveys to 38% of the full-time employees (N = 460) as our potential participants. During the process, voluntary participation and confidentiality were ensured. At the first round (T1), the respondents were requested to rate their servant leadership, coworker support, and their demographic information. In the second round (T2), 6 weeks after T1, the respondents were asked to rate their organization-based PO. In the third round (T3), 6 weeks after T2, the employees' level of knowledge hiding was rated by their coworkers. The respondents filled up the surveys anonymously. Each employee and supervisor were provided with a unique ID number randomly at the very beginning of the data collection process and they were requested to provide the ID number each time they participated in the survey, we could file it along with their responses from the previous rounds. Later, the researchers organized a set of two surveys, i.e., one for the subordinates and one for the supervisors using similar IDs for the pairing of received responses. Questionnaires were first administrated to 460 respondents during work time of which 356 responded to the T1 survey, yielding a response rate of 77.4%. Of these 356 respondents, 324 responded to the T2 questionnaire yielding a 91% response rate, and at T3 we obtained knowledge hiding ratings from the supervisors of 286 employees, yielding a response rate of 88.2%. Our results show that the final sample consisted of 62% of male respondents. The average age of the respondents was 38.2 years with 7.4 years of job experience and average educational qualification of postgraduation.

### Measures

All scales used in this research were measured on a five-point Likert scale ranging from 1 (strongly disagree) to 5 (strongly agree).

Servant leadership: To measure servant leadership, we used a seven-item scale developed by (Liden et al., 2015). We requested participants to rate the servant leadership of their immediate supervisor. Sample item: “My supervisor puts my best interests ahead of his/her own.” The Cronbach alpha value for this scale was 0.86.

Psychological ownership: This variable was measured using a six-item scale developed by Van Dyne and Pierce (2004). Sample item: “This is my organization.” The Cronbach alpha value for this scale was 0.74.

Perceived coworker support (PCS): To measure participants' level of PCS, we employed a three-item scale developed by Susskind et al. (2003) and used by Yang et al., 2020. Sample item: “When performing my job, I rely heavily on my coworkers”. The PCS scale's Cronbach alpha value was 0.72.

Knowledge hiding: A 12-item scale was adopted from Connelly et al. (2012), to measure KH. This scale measured employees' subjective judgment of their coworkers' knowledge hiding from them, the scale consisted of four items each for evasive hiding, playing dumb, and rationalized hiding. The scale opened with the following explanation: “For a moment, visualize in your mind your coworker; how does he/she behave upon receiving a request from you for any specific knowledge?” A sample item for determining playing dumb: “He/she pretends that he/she did not understand my request.” A sample item for determining evasive hiding: “I offered him/her some other information instead of what he/she wanted.” A sample item for determining rationalized hiding: “I told him/her that top management would not let anyone share this knowledge.” Following previous studies (e.g., Arain et al., 2019, 2021), we treated HK as a single construct. The model fit scores for this second-order confirmatory factor analysis was (χ^2^/df = 1.74, CFI = 0.94, TLI = 0.93, RMSEA = 0.05) and the Cronbach alpha score for this second order scale was (0.76).

Control variables: The demographic variables were included in the survey to statistically control their influences on the dependent constructs to rule out the clarifications for significant relationships. Following previous studies, such as Lam et al. (2009), Raja and Johns (2010), and Hameed et al. (2017), four demographic variables, i.e., gender, education, age, and experience (in the current organization) were included in the survey.

## Results

To assess the goodness of the model fit of the data, we used the following indices (Byrne, 2013): χ^2^/df, the comparative fit index (CFI), the Tucker-Lewis index (TLI), and the root-mean-square error of approximation (RMSEA). Hair et al. (2010) suggests an acceptable model should have CFI and TLI scores above 0.90 and an RMSEA value below 0.08. Following Bentler and Bonett (1980) we performed a series of CFA to compare the fit indices of the retained four-factor model with alternative models. Specifically, we compared the fit indices of the retained four-factor model (i.e., Model-1) with the two-factor (i.e., Model-2), and single-factor alternative model (i.e., Model-3). The results are provided in Table 1 and reveal that the retained four-factor model had a better fit to the data than the alternative models. Thus, these results (see Table 1) established the factorial validity of the hypothesized four-factor model, which we carried forward to conduct the hypothesis testing in SPSS.

**Table 1:** Comparisons of the CFA Results.

**Variables**	***χ^2^***	***df***	**CFI**	**TLI**	**RMSEA**
Model-1: the hypothesized four-factor model (i.e., servant leadership, psychological leadership, PCS, and knowledge hiding)	789.85	428	0.93	0.94	0.05
Model-2: the alternative three-factor model (psychological ownership and knowledge hiding were combined)	984.10	492	0.83	0.79	0.08
Model-3: the alternative one-factor model (all items were loaded onto a single factor)	1,343.63	319	0.67	0.63	0.12

N = 318; CFI, Comparative Fit Index; TLI, Tucker–Lewis index; RMSEA, root mean square.

The error of approximation.

Table 2 presents the means, standard deviations (SDs), and bivariate correlations of all the constructs. The results showed that most of the study's constructs were significantly correlated with each other.

**Table 2:** Descriptive statistics, Cronbach alpha, and correlations.

**Variables**	**M**	**SD**	**1**	**2**	**3**	**4**	**5**	**6**	**7**	
1. Gender	1.44	0.49	–							
2. Age	2.73	1.37	−0.035	–						
3. Education	1.56	0.73	−0.106	0.104	–					
4. Experience	2.55	0.88	−0.079	0.023	0.242**	–				
5. Servant Leadership	1.98	0.51	0.158	−0.027	−0.180	−0.114	*(0.86)*			
6. Psychological Ownership	2.20	0.81	0.115	0.029	−0.030	−0.018	0.475**	*(0.74)*		
7. Perceived coworker support	3.86	0.55	−0.031	−0.017	0.103	−0.030	0.447**	0.429**	*(0.72)*	
8. Knowledge hiding	3.14	0.74	−0.119	−0.006	0.143*	0.108	−0.554**	−0.594**	−0.457**	*(0.76)*

N = 318,

^*^p < 0.05;

^**^p < 0.01.

### Common method bias (CMB)

In the current research, CMB was analyzed through Harman's single factor test. After categorizing all the items into four factors, the results illustrated that the first factor explained only 28.2% of the variance. Thus, CMB was not a serious problem.

### Hypotheses testing

**Figure 2:**
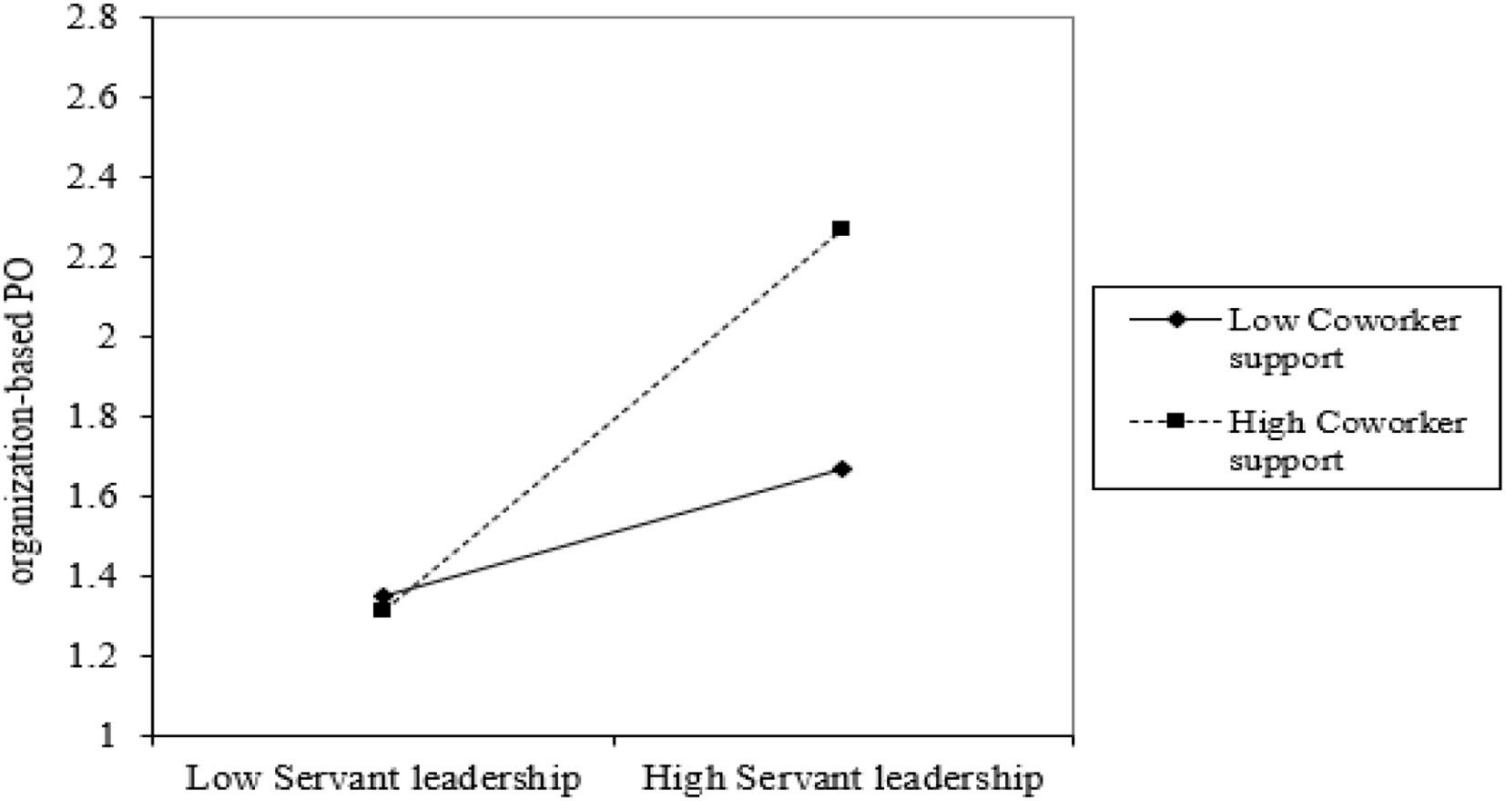
Interaction of SL and coworker support on organization-based PO.

To test our hypotheses we used the PROCESS macro for SPSS (Hayes, 2013) with 5000 bootstrap samples (MacKinnon et al., 2012). According to Hayes (2013), this macro was both appropriate and useful for computing interaction effects. The results in Table 3 show that SL was negatively related to KH (β = −0.18, SE = 0.06, *p* < 0.01), supporting hypothesis 1. Our results illustrated that SL was positively related to employees' PO (β = 0.60, SE = 0.05, *p* < 0.01), these results support hypothesis 2. The results also showed that PO was negatively related to KH (β = −0.45, SE = 0.05, *p* < 0.01), thus hypothesis 3 was supported. For mediation the indirect effect was significant with the 95% CI excluding zero, that is β = −0.12, with CI (−0.10, −0.08). Our hypothesis 5 indicated that PCS moderates the relationship between SL and PO. The results in Table 3 exhibited a significant interaction (SL × PCS) in the mediator model, which indicates that PCS moderated the relationship between SL and PO. These findings supported hypothesis 5. Figure 2 illustrates this relationship.

**Table 3:** Moderated mediation analysis for coworker support moderation, po mediation of servant leadership, and knowledge hiding.

	**Mediator variable PO model**			**Dependent variable KH model**		
**Predictors**	**B**	**SE**	**t**	**B**	**SE**	**t**
Servant leadership-Time1	0.32**	0.05	5.89	−0.18**	0.06	−3.20
Coworker support-Time1	0.14**	0.04	3.26			
SL × coworker support	0.16**	0.04	3.79			
PO-Time2				−0.45**	0.05	−8.17
Gender	0.08	0.08	1.05	−0.03	0.08	−0.32
Age	0.02	0.03	0.78	−0.02	0.03	−0.70
Education	0.12	0.05	2.25	0.38	0.06	6.51
Experience	0.06	0.04	1.28	0.07	0.04	1.52
R^2^		0.51				0.48
	Conditional indirect effects at specific value of moderator coworker support and independent variable (servant leadership): ±1 SD					
						95%
Dependent variable	Coworker support	Conditional indirect effect		SE	Lower	Upper
Knowledge Hiding-Time3	−1 SD (2.68)	−0.14		0.04	−0.20	−0.10
	+1 SD (3.80)	−0.08		0.04	−0.04	−0.08

## Discussion

This study aimed to examine how servant leadership could help organizations reduce knowledge hiding by enhancing the sense of organization-based psychological ownership in employees. We also investigated coworker support as an important resource that can help organizations to create psychological ownership in their employees which, in turn, can help them to overcome the intention to hide knowledge. Our results indicated that servant leadership positively influences psychological ownership. We also found that psychological ownership mediates the effect of servant leadership on knowledge hiding. Moreover, coworker support moderates the relationship between servant leadership and psychological ownership.

### Theoretical implications

The current research contributes to the leadership and knowledge management literature in several ways. First, this research adds to the understanding of the impact of positive leaders' behaviors in minimizing KH in the workplace. The existing studies on leadership and knowledge management have focused on studying positive knowledge behaviors (e.g., knowledge sharing) (Trong Tuan, 2017; Le and Lei, 2018). In the current study, we found that SL was negatively related to employee KH hiding, which has never been studied, except by Men et al. (2020) where ethical leadership was found to be negatively correlated to employee KH. Second, our findings indicate that SL positively influenced employees' sense of psychological ownership, which helps to minimize KH. The empirical results of this research extend strong support for the proposition that servant leaders who adopt an employee-centered management approach, stressing personal integrity and care for employees significantly affect employee attitudes and behaviors. Consistent with SLT, we argue that when subordinates think that they are working with servant leaders who not only display normatively appropriate behavior but also inspire and motivate their subordinates by demonstrating attractive characteristics (Mayer et al., 2012), then the subordinates are likely to develop a sense of psychological ownership, which leads to a reduction in KH. These findings are consistent with previous studies which highlighted that positive leadership could reduce employees' negative workplace outcomes by enhancing their sense of PO (Kim and Beehr, 2017). Furthermore, by examining the PO as a mediating mechanism between leadership and employee KH, this study responded to the call for more research by Men et al. (2020) who suggested that organization-based PO may help to reduce KH. Finally, we found that coworker support moderated the positive relationship between SL and employee organization-based PO. Our results demonstrated that a higher perception of coworker support strengthened the SL-employee PO relationship. We argue that employees will have more sense of PO if they believe that their work environment is supported by their colleagues. These findings are also consistent with previous research by Yang et al. (2020) which reported that when workers perceive adequate support from their coworkers, they generated positive emotional belongingness with the organization.

### Practical implications

The current research also offers some implications for managers and organizations. First, this study suggests that when managers demonstrate caring and selfless behavior toward their subordinates, it helps to reduce their negative behaviors (e.g., KH). Moreover, managers can perform a significant role in discouraging their subordinates' KH behaviors by promoting service-oriented behaviors (Liden et al., 2014) and serving as good role models. Managers could do so by accentuating personal integrity and care for their subordinates (Liden et al., 2008). Second, we suggest that by using the SL style in educational institutes, managers can develop a sense of PO in their subordinates as a result they may be less likely to hide knowledge from their colleagues. This indicates that organizations should select and recruit individuals for leadership positions who possess a set of skills that includes integrity and selflessness. Additionally, organizations should provide training to managers to use SL supportive behaviors to create a service-oriented environment that can help them to discourage employees' negative workplace behaviors. Lastly, organizations should promote SL behaviors through performance evaluation and rewarding programs to encourage managers to practice positive behaviors, which in turn reduce workers' KH behaviors.

## Limitations and future research directions

Like other studies, this research also has several limitations. First, the use of single-source data may raise concerns about common method bias. We think that coworker support and a sense of psychological ownership are perceptual measures that should best be taken by self-assessments. In the present research, the immediate supervisors rated employees' knowledge hiding, which allows using an additional source of data to strengthen our results. Second, we acknowledge that this research is conducted in the Pakistani context, which may limit the generalizability of the findings to other contexts. Future studies can collect data from other countries, which may provide greater validity through a generalization of the findings. Third, this study used a cross-sectional research design to obtain the data for hypothesis testing, which may impede us from exploring the causal relationships among variables. Therefore, it is suggested that future studies should validate the findings using a longitudinal research design because according to Li et al. (2015) longitudinal designs are considered more accurate for empirical data collection. This research employed a quantitative design to test the relationship between the hypotheses where future studies can employ qualitative attributes. This approach may provide a better opportunity for an in-depth and richer understanding of how servant leadership and support from colleagues and a sense of psychological ownership can help organizations to overcome the prevalence of knowledge hiding.

The findings of this research provide several directions for future research in the field of leadership and knowledge hiding. Among these, we suggest two interesting opportunities: first, this study recommends that future research should investigate other leadership styles (such as shared leadership) of psychological ownership which help managers and organizations to minimize knowledge hiding. Second, future studies can use other mediating mechanisms (e.g., person organization-fit) to explain the relationship between leadership and negative workplace outcomes.

## Data availability statement

The original contributions presented in the study are included in the article/supplementary material, further inquiries can be directed to the corresponding author.

## Ethics statement

Ethical review and approval was not required for the study on human participants in accordance with the local legislation and institutional requirements. Written informed consent from the patients/participants was not required to participate in this study in accordance with the national legislation and the institutional requirements.

## Author contributions

SA: conceptualization, data collection, methodology, analysis, writup, review, and editing final draft. LJ: supervision, review, and editing. All authors contributed to the article and approved the submitted version.

## Conflict of interest

The authors declare that the research was conducted in the absence of any commercial or financial relationships that could be construed as a potential conflict of interest.

## Publisher's note

All claims expressed in this article are solely those of the authors and do not necessarily represent those of their affiliated organizations, or those of the publisher, the editors and the reviewers. Any product that may be evaluated in this article, or claim that may be made by its manufacturer, is not guaranteed or endorsed by the publisher.
